# Environmental stress and nanoplastics’ effects on *Ciona robusta*: regulation of immune/stress-related genes and induction of innate memory in pharynx and gut

**DOI:** 10.3389/fimmu.2023.1176982

**Published:** 2023-05-29

**Authors:** Rita Marino, Daniela Melillo, Paola Italiani, Diana Boraschi

**Affiliations:** ^1^ Biology and Evolution of Marine Organisms, Stazione Zoologica Anton Dohrn (SZN), Napoli, Italy; ^2^ Institute of Biochemistry and Cell Biology, National Research Council (CNR), Napoli, Italy; ^3^ China-Italy Joint Laboratory of Pharmacobiotechnology for Medical Immunomodulation (CNR, SZN, SIAT), Shenzhen, China; ^4^ Laboratory of Inflammation and Vaccines, Shenzhen Institute of Advanced Technology (SIAT), Chinese Academy of Sciences, Shenzhen, China

**Keywords:** nanoplastics, *Ciona robusta*, innate immunity, innate memory, stress

## Abstract

In addition to circulating haemocytes, the immune system of the solitary ascidian *Ciona robusta* relies on two organs, the pharynx and the gut, and encompasses a wide array of immune and stress-related genes. How the pharynx and the gut of *C. robusta* react and adapt to environmental stress was assessed upon short or long exposure to hypoxia/starvation in the absence or in the presence of polystyrene nanoplastics. We show that the immune response to stress is very different between the two organs, suggesting an organ-specific immune adaptation to the environmental changes. Notably, the presence of nanoplastics appears to alter the gene modulation induced by hypoxia/starvation in both organs, resulting in a partial increase in gene up-regulation in the pharynx and a less evident response to stress in the gut. We have also assessed whether the hypoxia/starvation stress could induce innate memory, measured as gene expression in response to a subsequent challenge with the bacterial agent LPS. Exposure to stress one week before challenge induced a substantial change in the response to LPS, with a general decrease of gene expression in the pharynx and a strong increase in the gut. Co-exposure with nanoplastics only partially modulated the stress-induced memory response to LPS, without substantially changing the stress-dependent gene expression profile in either organ. Overall, the presence of nanoplastics in the marine environment seems able to decrease the immune response of *C. robusta* to stressful conditions, hypothetically implying a reduced capacity to adapt to environmental changes, but only partially affects the stress-dependent induction of innate memory and subsequent responses to infectious challenges.

## 1 Introduction

It is well known that marine organisms ingest plastic particles, including fish (1, 2) and several invertebrate species (3–5). The major threat of plastics released into the environment is related to the degradation of these materials into micro- and nano-sized particulates that can more easily bioaccumulate (6). Across marine filter-feeding invertebrate organisms, the solitary ascidian *Ciona robusta* represents an optimal model to study the impact of sub-micron particle bioaccumulation on fundamental physiological functions (7, 8). In fact, unlike bivalves (9), *C. robusta* does not have the ability to sort particles and reject the unsuitable material (10). The impact of nanoplastics accumulation on the immune defensive functions is of particular interest because of the importance of such functions for optimal adaptation to environmental changes. To fully understand the possible impact of nanoplastics on *C. robusta* it is therefore important to assess it in conditions of environmental stress.

Major stressful environmental conditions for ascidians are starvation and hypoxia. Starvation is known to affect the ascidian metabolic profile and induce autophagy-related genes. In a metabolomic study on *Halocynthia roretzi*, a strong upregulation of defence and energy metabolites was observed in response to starvation, largely mediated by the impact on the gut associated microbiota (11).

Hypoxia is a decrease in dissolved oxygen that causes significant physiological disturbances in many marine organisms (12, 13), including increased vulnerability to diseases and parasites (14). Hypoxia threshold is species- and stage-specific (13), and its effects depend on the presence of other environmental challenges (warming, acidification, pollution). Hypoxia causes a decrease of energy supply from mitochondrial metabolism, which cells seek to compensate by undergoing a metabolic reprogramming (*i.e.*, enhanced glycolysis, glutaminolysis, fatty acid synthesis and decreased gluconeogenesis, nucleotide synthesis, fatty acids β-oxidation) that mostly involves the family of hypoxia-inducible transcription factors (HIFs) (15). In mammalian immune cells, hypoxia and HIF signalling influence immune cell functions in a cell-type specific manner (16). Of special interest is the cross-talk between hypoxia and inflammation. Inflammation plays a key role in the physiological response to hypoxic stress, as shown for instance by the increase of circulating inflammatory cytokines in individuals with mountain sickness (17). Conversely, tissue sites where an inflammatory reaction takes place undergo significant shifts in metabolic activity leading to O_2_ deficiency, defined as “inflammatory hypoxia” (16, 18). In addition, HIF transcription/stabilisation can be activated by a variety of inflammation-related extracellular factors, such as bacterial products (*e.g.*, lipopolysaccharide or LPS), TNF-α, IL-1, reactive oxygen and nitrogen species (ROS, RNS), even in normoxic conditions.

In the present study, we have evaluated the primary response of *C. robusta* in terms of expression of immune and oxidative stress-related genes induced by exposure to a combined hypoxic and starvation stress, and how the presence of nanoplastics in the environment can interfere with the stress-induced immune adaptation response. We focused on two organs involved in immune defence, namely the pharynx and the gut, as these organs, involved in respiration and digestion, are directly exposed to environmental stresses. We have also evaluated how a previous exposure to such combined stresses could influence the ability of the *C. robusta* immune organs to react to a subsequent bacterial challenge.

## 2 Materials and methods

### 2.1 Animals and treatments

Adults of *C. robusta* were collected in the small sea of Taranto, Italy, and maintained at the SZN in tanks with circulating aerated seawater at 18°C with proper feeding. Hypoxia/starvation (H/S) treatment was performed by starving animals in tanks for 12 h, then transferring individual animals in 200 mL millifiltered sea water (MFSW) within a 250 mL glass beaker at 18°C for 2 or 18 h (starvation plus hypoxia). Treatments with polystyrene beads (MPs) of either 0.1 µm or 0.35 µm (cat. 00876 and 07306; Polysciences, Inc., Warrington, PA, USA) was conducted by diluting nanoplastic beads in MFSW at the concentration of 9.1x10^8^ particles/mL and 7.4x10^7^ particles/mL, respectively, and adding them to individual animals in beaker for 2 or 18 h. The concentration of 0.1 µm nanoplastics was selected based on previous dose-response experiments as the concentration causing the highest bioaccumulation after 2 h of exposure (8, and data not shown). The concentration of 0.35 µm nanoplastics was selected in order to correspond to that of 0.1 µm particles in terms of surface area. Endotoxin treatment was carried out by inoculation of 50 μg of lipopolysaccharide (LPS, from *Escherichia coli*, serotype O55:B5; Merck Sigma-Aldrich^®^, St. Louis, MO, USA). LPS was inoculated in 50 μL of marine solution (MS: NaCl 0.45 M, MgCl_2_ 26 mM, KCl 11 mM, CaCl_2_ 12 mM, pH 7.4), through the tunic between the two siphons.

For memory experiments, animals were first exposed to H/S stress alone or in the presence of nanoplastics for 2 or 18 h as described above, then were transferred to the aquarium, where they were kept in large aerated tanks and properly fed for additional 7 days (resting or extinction period). After the resting period, animals were injected with 50 μL of LPS, as described above. After 24 h, animals were sacrificed and exanguinated, and gut and pharynx fragments were collected for gene expression analysis. For each condition, three animals were included.

### 2.2 RNA extraction and real-time PCR

Tissue samples were weighed and immediately homogenised with an Ultra-Turrax T25 at 0°C with 3 cycles of 30 s, then processed for total RNA extraction with commercially available kits (miRNeasy Kit; Qiagen, Hilden, Germany), according to the manufacturer’s instructions. A mix of Oligo (dT) and random-primed single stranded cDNA were synthetised from 2 μg of pharynx RNA using the QuantiTect Reverse Trascription Kit (Qiagen).

Real-time PCR experiments were carried out with a RotorGene instrument (Qiagen) with RealAmp qPCR Master mix chemistry (GeneAll Biotechnology Co., Ltd., Seoul, South Korea). Specific primers were designed, according to the nucleotide sequence, for genes encoding the *C. robusta* homologues of the complement component C3-1 (*C3-1*), the C3 receptor C3aR (*C3ar*), the two isoforms of interkeukin-17 *Il17-1*, *Il17-2*, the IL-17 receptor *Il17r*, the tumour necrosis factor *Tnf*, the transforming growth factor beta *Tgfb*, the LPS binding protein *Lbp*, the Toll-like receptors *Tlr-2* and *Tlr13*, the cluster of differentiation 36 *Cd36*, the variable chitin-binding proteins *VCBP-B* and *VCBP-C*, the superoxide dismutase A *SodA*, the glutathione-S transferase *GST* and the glutathione reductase *GR* (**Table 1**). Gene nomenclature is designed according to previous publications (19–27) and includes the indication of the *C. robusta* gene isoforms as a number after a dash. Other isoforms of C3 (*C3-2*), IL-17 (*Il17-3*), and TLR (*Tlr-1*) could not be evaluated because their expression resulted undetectable in every condition in both gut and pharynx. Likewise, expression of the gene encoding the precursor of the enzyme phenoloxidase was not detectable. After preliminary evaluation of different housekeeping genes (*Actin*, *S27*, *Gapdh*), the glyceraldehyde 3-phosphate dehydrogenase gene *Gapdh* was selected for its consistent expression stability, and used as reference gene in all experiments. All primers produced single-band amplicons of the expected size, which were verified by DNA sequencing. Reactions were performed in triplicate, and the PCR programme included a denaturation step (95°C for 15 min) followed by 40 cycles of amplification (94°C for 15 s, 60°C for 30 s, and 72°C for 30 s), and a final extension step (72°C for 10 min). PCR amplification efficiencies, calculated for primer pairs of the reference and target genes, were both 2. All data were normalised against *Gapdh* using the Pfaffl method (28). Real-time PCR results are reported as relative gene expression towards *Gapdh* or as the ratio between treated and control animals.

**Table 1:** List of primers used for evaluating gene expression in *C. robusta*.

GENE	FORWARD	REVERSE	Genbank acc. No.
*C3-1*	5′ -acagacgtggcgtgtgcaag-3	5′-tactttgcctaggaggccggt-3′	AJ320542
*C3ar*	5′ -ttgccccgccatgcgagga-3′	5′-aggtacgactccatacaacacc-3′	AJ966353
*Il17-1*	5’-ccgggaacgtgacagaaaac-3’	5’-tcgtggaagcaccataggga-3’	NM_001129875.1
*Il17-2*	5′-cgggtgcattgcttctagt-3′	5′-cacgcaggtacagcctattg-3′	NM_001129874.1
*Il17r*	5′-gtgacccgtggcaatcaatgg-3′	5′-caagttaggcattttgctccgt-3′	AY261862
*Tnf*	5′-catctccccaccctactacac-3′	5′-atttgcgcaaacgtctggca-3′	AM982527
*Tgfb*	5′-ctcgttcaaatgtgtctcaaaccg-3′	5′-cgttgccagattttacgacg-3′	AB210727
*Lbp*	5′-ggtttcgggaagctgggatt-3′	5′-gaaggggcctgtttcttcca-3′	XM_002126995.2
*Tlr-2*	5′-acgcaagaaacaagagagacg-3′	5′-gcttttcttccatttcctccagc-3′	AB495262.1
*Tlr13*	5′-cggaagcattgtgctggaaa-3′	5′-acgcaagacaaatacgcctg-3′	XM_002120484.4
*Cd36*	5′-ggttcgcttttatttcttggacct-3′	5′-ctgcaccgtttggtttacgg-3′	XM_009860510.1
*VCBP-B*	5’- ttcacccacacggagattgg-3’	5’-cggcgcttgatctggatact-3’	HQ324165
*VCBP-C*	5’-gcaacactcagtggcaaca-3’	5’-ccgcatttctcatctcgcac-3’	NM_001204050
*SodA*	5’- ccacaaaatatagacgaaggcgac-3’	5’-gacaacgcactattcaacggg-3’	XM_002121064
*GST*	5’-ccaagcgatgctaatgcgag-3’	5’- cggcgggattgaggtatgt-3’	XM_002121841
*GR*	5’- agcacttcttacaccagttgc-3’	5’-cccaatgggtggatgactga-3’	XM_002119519
*Gapdh*	5′-cattttcgacgcaggagctg-3′	5′-ctgcgtggtgtttaactggc-3′	XM_002131188.4

### 2.3 Statistical analysis

All values were expressed as mean ± SEM of samples from 3 animals with the same treatment. Statistical significance of differences between treatments was assessed by using the one sample Student’s *t* test followed by non-parametric Mann-Whitney U-test correction for PCR data of primary effects, and ordinary one-way ANOVA of one sample *t* test for PCR data of memory experiments, using the GraphPad Prism 6 software. *p* values <0.05 were considered statistically significant.

## 3 Results

### 3.1 Induction of immune response by hypoxia/starvation stress

The basal expression of sixteen genes was examined in *C. robusta*. Genes were selected based on three criteria, immune-related genes that are already known to be involved in the *Ciona* immune responses (*C3-1*, *C3ar*, *Il17-1*, *Il17-2*, *Il17r*, *Tnf*, *Tgfb*, *Lbp*, *Tlr-2*, *Tlr13*, *Cd36*) (19–26), pharynx- and gut-specific genes involved in mucosal immunity (*VCBP-B* and *VCPB-C*) (27) and oxidative stress-related genes (*Sod-A*, *GST*, *GR*) (29). Gene expression was measured in the two organs that display immune reactivity, namely the pharynx and the gut. The relative contribution of haemocytes, the main circulating immune cells of *C. robusta*, was not specifically assessed, as the number of recovered cells was insufficient for RNA extraction.

Results in **Table 2** show the basal expression of immune/stress-related genes in both organs in unexposed animals kept in optimal conditions of oxygenation and nutrition. Gene expression was also measured in animals treated with the bacterial agent LPS, to mimic exposure to an infectious challenge. Substantial variations in gene expression could be observed, which differ between the two organs. In the pharynx, reaction to LPS resulted in a general upregulation of the majority of the immune/stress-related genes, with a very high increase in the expression of *Tnf*, *Tgfb*, *VCBP-B* and *VCBP-C* and a significant but less substantial increase of *Il17-2* and *Tlr-2* expression. Only one gene, *GST*, was significantly down-regulated in the pharynx in response to LPS. The scenario in the gut is very different, with only one gene, *C3ar*, significantly up-regulated in response to LPS. The expression of most genes is either unchanged or down-regulated by LPS in the gut, although a statistically significant decrease was only observed for *Il17r*, *VCBP-B* and *GR*. Notably, expression of *VCBP-B*, which was increased over 4000x in the pharynx, was completely inhibited by LPS in the gut (**Table 2**).

**Table 2:** Immune/stress-related gene expression levels in the pharynx and gut of *C. robusta*.

Gene	Gene expression level ^a^ (mean ± SEM)			
	Pharynx		Gut	
	Control	LPS	Control	LPS
*C3-1*	2.52 ± 1.49	4.54 ± 1.22	1.22 ± 0.50	0.10 ± 0.05
*C3ar*	2.29 ± 1.73	2.18 ± 0.59	1.04 ± 0.21	6.55 ± 1.08^*^
*Il17-1*	1.16 ± 0.47	1.28 ± 0.38	1.08 ± 0.29	0.53 ± 0.21
*Il17-2*	1.76 ± 1.16	10.02 ± 3.67^**^	1.49 ± 0.94	1.30 ± 0.19
*Il17r*	1.36 ± 0.61	1.37 ± 0.49	1.09 ± 0.33	0.03 ± 0.01^*^
*Tnf*	1.19 ± 0.40	62.3 ± 5.7^**^	1.18 ± 0.51	0.08 ± 0.02
*Tgfb*	1.06 ± 0.24	80.9 ± 9.5^**^	2.12 ± 1.37	0.52 ± 0.24
*Lbp*	1.05 ± 0.23	1.90 ± 0.17	1.10 ± 0.35	0.31 ± 0.09
*Tlr-2*	1.31 ± 0.53	11.37 ± 1.41^*^	1.08 ± 0.26	0.62 ± 0.01
*Tlr13*	1.04 ± 0.20	0.45 ± 0.14	1.35 ± 0.74	0.28 ± 0.04
*Cd36*	1.06 ± 0.24	0.59 ± 0.08	1.17 ± 0.49	0.15 ± 0.05
*VCBP-B*	1.11 ± 0.36	4129.5 ± 1042.5^**^	1.25 ± 0.61	0.00 ± 0.00^*^
*VCBP-C*	1.09 ± 0.27	30.24 ± 6.35^*^	1.13 ± 0.40	0.63 ± 0.09
*SodA*	1.04 ± 0.20	0.82 ± 0.59	1.43 ± 0.79	0.50 ± 0.15
*GST*	1.06 ± 0.26	0.03 ± 0.00^**^	2.43 ± 1.93	0.17 ± 0.02
*GR*	1.09 ± 0.33	3.05 ± 1.38	1.26 ± 0.51	0.06 ± 0.00^*^

a relative to Gapdh.

^*^p<0.05, ^**^p<0.01, LPS vs. controls.

**Figure 1:**
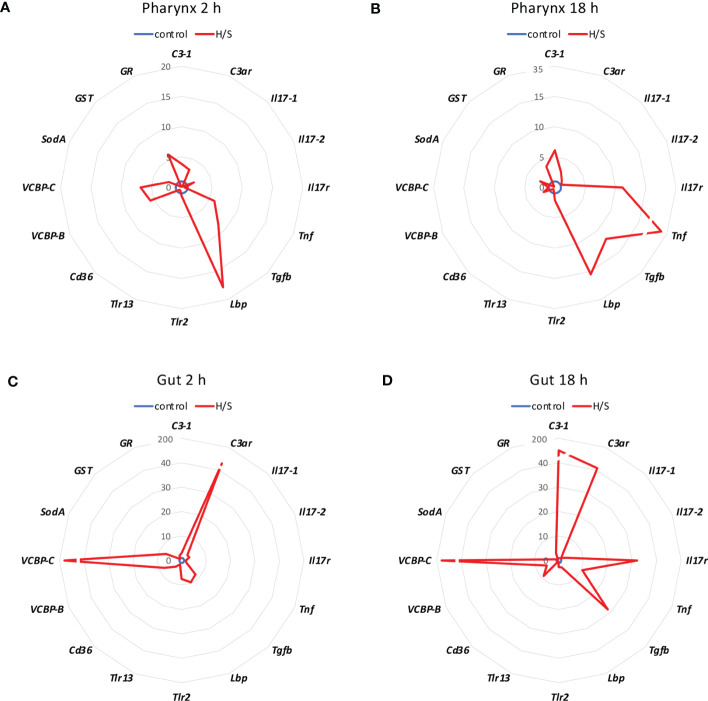
Hypoxia/starvation stress-induced gene expression in the pharynx and gut of *Ciona robusta.* Gene expression was measured in the pharynx and in the gut of animals exposed to hypoxia/starvation (H/S) stress (red line) for 2 h or 18 h. **(A, B)** gene expression in the pharynx; **(C, D)** gene expression in the gut. **(A, C)** gene expression at 2 h; **(B, D)** gene expression at 18 h. Data are the mean of values from 3 animals and are expressed relative to gene expression in control naïve animals (blue line). The mean values of gene expression relative to the housekeeping gene *Gapdh*, the SEM values and the statistical analysis are reported in the **Supplementary Table S1**.

We analysed whether stress conditions could induce an immune reaction similar to exposure to an infectious agent. Stress was obtained by keeping animals in a small volume of millifiltered seawater (MFSW) for 2 or 18 hr, without oxygenation and feeding. The expression of the selected immune/stress-related genes was examined in the stressed animals as compared to control animals kept in oxygenated tanks with food. The results in **Figure 1** show that the stress induced by hypoxia/starvation (H/S) has a significant effect on the expression of immune/stress-related genes, and that this effect is different depending on the organ and the duration of stress. In the pharynx, a short H/S stress induced a significant upregulation of *C3-1*, *Tnf*, *Tgfb*, *Lbp*, *VCBP-B*, *VCBP-C* and *GR* (**Figure 1A**). An H/S stress of 18 h induced expression of the *Il17r* gene and further increased *Tnf* expression, while upregulation of *C3-1, Tgfb* and *Lbp* remained sustained at the same level, and that of *VCBP-B* and *VCBP-C* returned to baseline (**Figure 1B**). The response of the gut to H/S stress was substantially different. The short 2 h stress induced a strong up-regulation of the *C3ar* and *VCBP-C* genes, and a significant increase of *Il17-2, Tgfb*, *Lbp*, *Trl-2, VCBP-B* and *SodA*, while other genes were only marginally affected (**Figure 1C**). After 18 h of H/S stress, expression of *C3-1*, *Il17r*, *Tnf* and *Cd36* was up-regulated, up-regulation of the *C3ar, VCBP-B* and *VCBP-C* was sustained at the same level, while that of *Tgfb* was further increased, and that of the other genes decreased towards basal expression levels (**Figure 1D**). Full data (mean levels of gene expression with SEM and statistical analysis) are reported in the **Supplementary Table S1**. Thus, H/S stress could induce substantial variations in immune/stress gene expression both in the pharynx and in the gut, suggesting an active immune adaptation to the environmental changes. The kinetic differences in the expression profiles underline the evolution of the adaptive changes, while the difference between the two immune organs strongly support their distinct roles in immune responses.

### 3.2 Modulation of stress-induced immune response by nanoplastics

**Figure 2:**
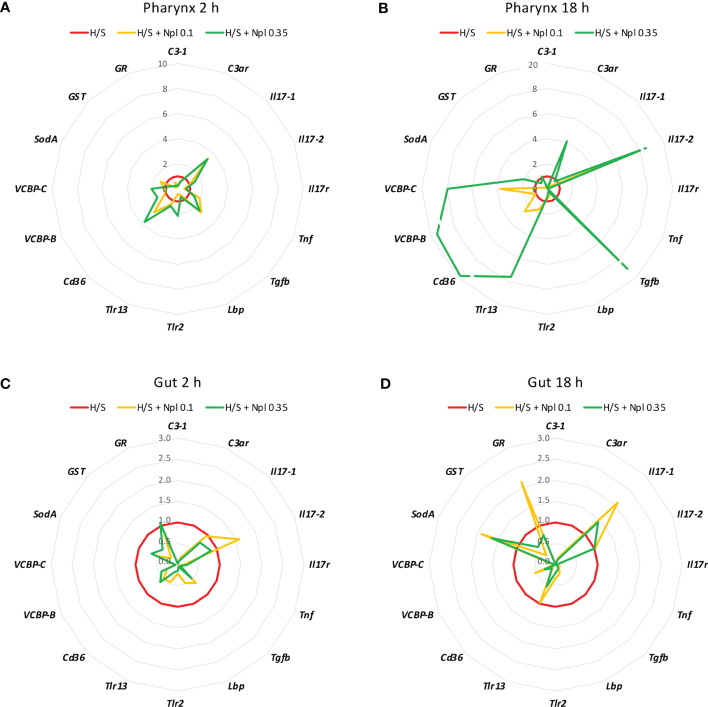
Effect of nanoplastics on the hypoxia/starvation stress-induced gene expression in the pharynx and gut of *Ciona robusta*. Gene expression was measured in the pharynx and in the gut of animals exposed to hypoxia/starvation (H/S) stress alone (red line) or combined with nanoplastics for 2 or 18 h. **(A, B)** gene expression in the pharynx; **(C, D)** gene expression in the gut. **(A, C)** gene expression at 2 h; **(B, D)** gene expression at 18 h. Nanoplastics of two different sizes were used, 0.1 μm (yellow line) and 0.35 μm (green line). Data are the mean of values from 3 animals and are expressed relative to gene expression in H/S-exposed animals. The mean values of gene expression relative to the housekeeping gene *Gapdh*, the SEM values and the statistical analysis are reported in the **Supplementary Table S2**.

**Figure 3:**
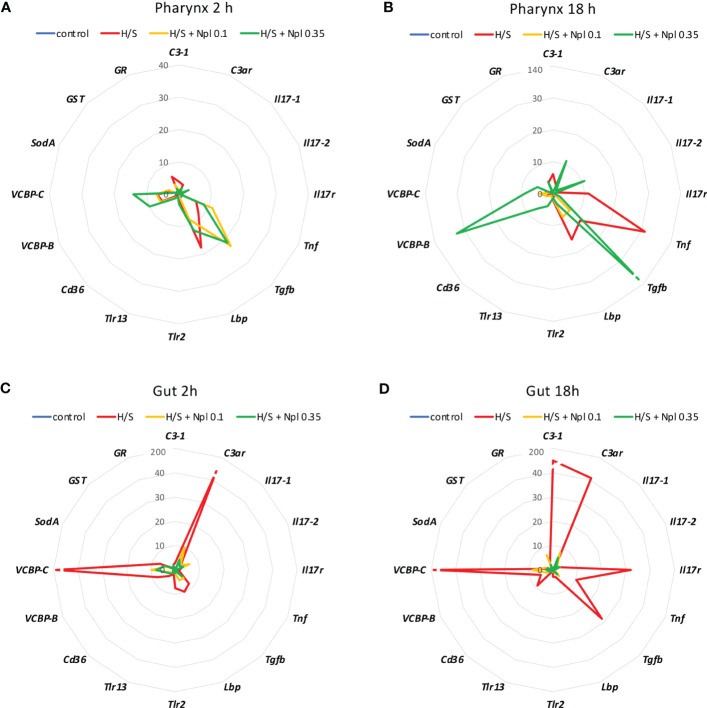
Effect of the combined exposure to hypoxia/starvation and nanoplastics on the immune/stress-related gene expression in the pharynx and gut of *Ciona robusta*. Gene expression was measured in the pharynx and in the gut of animals exposed to hypoxia/starvation (H/S) stress alone (red line) or combined with nanoplastics for 2 or 18 h. **(A, B)** gene expression in the pharynx; **(C, D)** gene expression in the gut. **(A, C)** gene expression at 2 h; **(B,D)** gene expression at 18 h. Nanoplastics of two different sizes were used, 0.1 μm (yellow line) and 0.35 μm (green line). Data are the mean of values from 3 animals and are expressed relative to gene expression in control naïve animals (kept in tank in optimal oxygenation and nutrition conditions; blue line). The mean values of gene expression relative to the housekeeping gene *Gapdh*, the SEM values and the statistical analysis are reported in the **Supplementary Tables S2** (pharynx) and **S3** (gut).

To assess whether concomitant exposure to nanoplastics could affect the stress-induced response, animals were exposed to polystyrene beads of two different sizes (diameter 0.1 and 0.35 µm) in H/S conditions. After 2 or 18 h, the gene expression was assessed. As shown in **Figures 2** and **3**, the presence of nanoplastics could affect the gene expression changes induced by H/S both in the pharynx and in the gut. In the pharynx, the presence of nanoplastics induced some significant changes in the gene expression induced by a 2 h exposure to H/S stress alone, *i.e.*, further up-regulation of H/S stress-induced *Tgfb* gene expression, and down-regulation of H/S stress-induced *C3-1* and *Lbp* gene expression (**Figure 2A**). Other changes (*e.g.*, up-regulation of the stress-induced decrease of *Il17-1* and *Cd36* genes) were not statistically significant. After 18 h, the presence of nanoplastics induced several changes compared to H/S alone, in particular a decrease in *C3-1*, *Il17r* and *Tnf* expression and an increase of *Il17-2* (**Figure 2B**). In the pharynx, differences were also noted between nanoplastics of different size, with the larger particles in combination with H/S for 18 h able to up-regulate the expression of *C3ar*, *Tgfb*, *Tlr13*, *Cd36*, *VCBP-B* and *VCBP-C* (not induced by the combination of H/S and small nanoplastics).

In the gut, no substantial size-dependent effect of nanoplastics was noted on the gene modulation induced by H/S stress (**Figures 2C, D**). After 2 h of exposure to H/S stress in the presence of nanoplastics, gene expression was either unaffected or decreased, with a significant inhibition of *C3-1*, *C3ar*, *Tgfb*, *Lbp*, *Tlr-2*, and *VCBP-C* (**Figure 2C**). After 18 h of combined exposure to H/S stress and nanoplastics, gene expression changes were more evident than with the shorter exposure, showing a significant decrease in the expression of *C3-1*, *C3ar*, *Il17r*, *Tnf*, *Tgfb*, *Cd36, VCBP-B* and *VCBP-C* (**Figure 2D**). The increased expression of *Il17-1*, *SodA* and *GR* genes in the presence of nanoplastics was not statistically different from H/S stress alone.

Notably, the comparison of the gene expression profiles induced by H/S stress and by H/S stress plus nanoplastics with those of naïve animals shows that the presence of nanoplastics may reduce some of the gene expression changes induced by H/S stress (**Figure 3**). This is particularly evident in the gut, both after a short or long exposure to stress, where exposure to nanoplastics substantially limited the up-regulation of several of the immune/stress genes induced by H/S (**Figures 3C, D**). Full data (mean levels of gene expression with SEM and statistical analysis) are reported in the **Supplementary Tables S2** and **S3**.

Thus, the presence of nanoplastics may dampen some of the stressful effects induced by H/S, which however may result in inadequate adaptation to the environmental changes.

### 3.3 Organ-specific modulation of immune/stress-related gene expression by stress-induced innate memory

**Figure 4:**
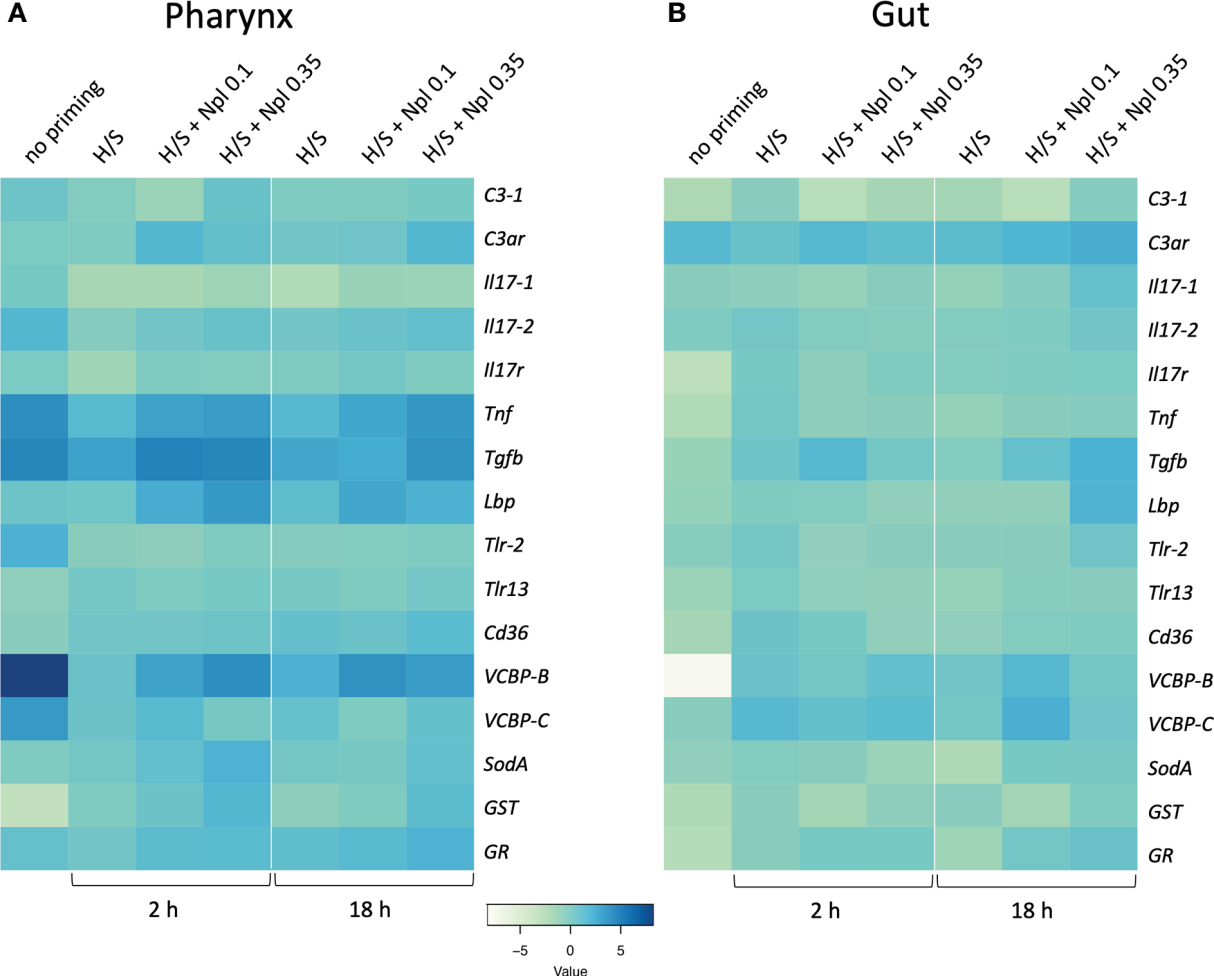
Stress-induced memory response to LPS in *Ciona robusta*. The response to LPS was measured in naïve animals (no priming) and animals pre-exposed to H/S stress alone (H/S) or with nanoplastics of 0.1 μm (H/S + Npl 0.1) and 0.35 μm (H/S + Npl 0.35) for 2 or 18 h. After pre-exposure, animals were then transferred in tanks and kept in optimal oxygenation and nutrition conditions for one week, before receiving 50 μg LPS intratunically. Gene expression in response to the LPS challenge was measured 24 h later. **(A)** gene expression in the pharynx; **(B)** gene expression in the gut. The heatmaps report the results of gene expression (mean values from 3 animals relative to gene expression in unchallenged control animals). The SEM values and the statistical analysis are reported in **Supplementary Tables S4** (pharynx) and **S5** (gut).

We assessed the response to the prototypical bacterial agent LPS in the pharynx and gut of animals that were previously primed by H/S in comparison to unprimed controls. As described above for the primary response, the “memory” response was measured as expression of immune/stress-related genes. As already mentioned, the response of naïve animals to LPS encompasses a general up-regulation of immune/stress-related genes in the pharynx, opposite to a general down-regulation in the gut (**Table 2**). In animals previously exposed to H/S stress (either 2 or 18 h, followed by one week in tank with proper oxygenation and nutrition) the response to LPS was significantly different, implying the establishment of a stress-induced innate memory, able to modulate the response to an infectious challenge. In the pharynx, pre-exposure to H/S stress induce a substantial decrease in the expression of several genes and the up-regulation of the *GST* gene (**Figure 4A**, **Table S4**). Notably, down-regulation exclusively occurred for genes that were up-regulated in response to LPS in naïve animals, while up-regulation occurred for the only gene that was strongly down-regulated by LPS in naïve animals. This implies that animals exposed to H/S stress have developed “tolerance” to LPS in the pharynx, as their expression profile of immune/stress-related genes is similar to the basal expression profile of untreated animals (**Table S4**). A similar induction of “tolerance” to LPS was observed in the gut of animals pre-exposed to H/S stress. While the response to LPS in the gut is a general down-regulation of immune/stress gene-expression, expression of these genes in response to LPS was higher in animals pre-exposed to H/S stress (**Figure 4B**, **Table S5**). Thus, as in the pharynx, pre-exposure to H/S stress induced a state of unresponsiveness to LPS in the gut, with the gene expression profile of H/S-primed animals challenge with LPS similar to that of naïve unchallenged animals (**Table S5**). No substantial differences were observed between 2 *vs*. 18 h of pre-exposure to H/S stress (**Figures 4A, B**).

### 3.4 Effect of nanoplastics on the organ-specific stress-induced innate memory

We examined whether the combined exposure to H/S stress and nanoplastics could affect the tolerance type of innate memory response to LPS induced by H/S stress alone. Data in **Figure 4** show the innate memory response to LPS of animals pre-exposed to H/S stress alone (H/S) or in the presence of small 0.1 µm nanoplastics (H/S + Nlp 0.1) or 0.35 µm particles (H/S + Npl 0.35), compared to unexposed animals (no priming). Notably, nanoplastics appear to reverse in some cases the gene down-regulation caused by pre-exposure to H/S stress in the pharynx (*e.g.*, *Tnf*, *Tgfb*, *VCBP-B*) and increase the expression of genes that were either up-regulated (*GST*) or not affected (*Lbp, SodA*) by H/S stress (**Figure 4A**, **Table S4**). Some changes were common to nanoplastics of both sizes, *e.g.*, upregulation of *Tnf* and *Lbp* gene expression, while other were size-specific, with larger particles able to significantly upregulate *SodA* and *GST*, while small particles were inactive (**Figure 4A**, **Table S4**). After 18 h, the nanoplastics effects on H/S-induced changes were essentially maintained, except for the loss of effects for small particles on H/S-induced down-regulation of *Tgfb* and the up-regulation of *GR* by large particles. Notably, up-regulation of *C3ar* was observed with larger particles (inactive at 2 h), while the effect of small particles (active at 2 h) was lost (**Figure 4A**, **Table S4**). In the gut, both common and size-dependent effects of nanoplastics were observed on the gene modulation induced by H/S stress (**Figure 4B**, **Table S5**). After 2 h of pre-exposure to H/S stress in the presence of nanoplastics, small particles could induce a statistically significant increase of the expression of the *Tgfb* gene compared to H/S alone, an effect not shared by larger particles (**Figure 4B**, **Table S5**), while after 18 h both types of particles were active (**Figure 4B**, **Table S5**). After 18 h, smaller particles were able to further increase the H/S-induced expression of the *VCBP-B* and *VCBP-C* genes, as opposed to the lack of effect by larger particles (**Figure 4B**, **Table S5**).

## 4 Discussion

Studies of inflammatory responses to LPS in tunicates have demonstrated an up-regulation of cytokine-like genes such as *Tnf* (25) and *Il17* (22), as well as complement components (30, 31), mostly evident in haemocytes and in the pharynx. On the other hand, data concerning the stress-induced response are poorly represented. Several invertebrate species, such as oysters, are remarkably resilient to fluctuating environmental conditions, and this adaptation is based on a large repertoire of immune-related genes, many of which are greatly expanded in the genome (32). Rather than on the main circulating immune cells, the haemocytes, in our study we focused on the pharynx and the gut, two organs involved in respiration and digestion and therefore directly exposed to environmental stresses, to assess the organ-specific immune adaptation. We found that an environmental stress, represented by hypoxia and starvation (H/S), up-regulates the expression of a variety of immune/stress-related genes, both in the pharynx and in the gut of *C. robusta*. Interestingly, beyond the up-regulation in both organs of the complement gene *C3-1*, the cytokine/cytokine receptor genes *Il17r*, *Tnf*, *Tgfb* and *Lbp*, the chitin-binding protein genes *VCBP-B* and *VCBP-C* and the stress-related gene *GR*, we could observe a gut-specific up-regulation of *C3ar*, *Tlr-2* and *CD36*. We also examined how the additional presence of another environmental stress, *i.e.*, nanoplastics, may affect the adaptation reaction to H/S. In the pharynx, co-exposure to H/S and nanoplastics induces an enhanced up-regulation of the *Il17-2*, *Tgfb*, *VCBP-B* and *VCBP-C* genes, a substantial down-regulation of *Tnf*, and no change in the other genes. On the contrary, the effect of nanoplastics co-exposure in the gut is a general down-regulation of all the immune/stress-related genes. These data suggest two scenarios. First, while the H/S stress strongly affects the expression of immune/stress-related genes, in line with the expected adaptation to the changing environmental conditions, such effects are different between the two immune organs, stressing their different immune protective role. The second observation is that the presence of nanoplastics interferes with the stress-induced immune response, which may therefore affect the adaptation capacity of the animals.

It is interesting to examine the regulation of the *Lbp* gene in response to LPS (used as positive control) *vs*. stress. In mammals, LBP is induced by LPS stimulation and displays a concentration-dependent modulation of LPS activity: at low concentrations, LBP shuttles LPS to monocytes or other immune cells, whereas at high concentration it inhibits the endotoxic activity of LPS by facilitating its elimination (33–35). Unexpectedly, in the present study we found that the *Lbp* gene in *C. robusta* is unresponsive to LPS stimulation in both organs, while it is up-regulated by H/S stress in particular in the pharynx. These results seem to suggest that *Lbp* activation is independent of LPS, but necessary in the adaptive immune reaction initiated by H/S stress. Notably, this protective effect is partially abolished in presence of nanoplastics, supporting the hypothesis that animals exposed to nanoplastics may be less able to adapt to environmental changes.

The induction of innate memory was examined in the pharynx and in the gut of animals previously exposed to H/S stress alone or together with nanoplastics. Pre-exposed animals were administered with LPS, and the expression of immune/stress-related genes was compared to that of naïve animals. Upon previous H/S and H/S nanoparticles experience, haemocytes and/or pharynx cells react to a systemic LPS challenge by turning off most immune/inflammatory response as compared to unprimed animals, a response that compatible with a tolerance type of innate memory, while only transcription of *GST* is increased in animals pre-exposed to H/S and nanoplastics. GSTs are a multigene family of isozymes that catalyse the conjugation of glutathione (GSH) to several molecules. In our animal model, we could hypothesise that immune memory in the pharynx is linked to the oxidative stress generated by hypoxic conditions, which may be exacerbated in the presence of nanoplastics. In fact, nanoplastics may amplify the oxidative and inflammatory effects of H/S by reducing the filtration rate of the animals and related O_2_ supply, by clotting mucus and clogging the pharynx stigmata. Upon challenge with LPS, known to lower the GSH levels in vertebrate models (36), there is a strong upregulation of the *GST* gene in animals pre-exposed to H/S and nanoplastics, likely underlying a memory-dependent protective response aiming at restoring the tissue GSH pool and its detoxification effects.

In order to appropriately assess the innate memory induced by H/S and nanoplastics in the gut, it is important to keep in mind some organ-related effects. The role of starvation in the gut is twofold. As in all tissues and organs, together with hypoxia, starvation contributes in eliciting oxidative and inflammatory stress. More specifically in the gut, starvation impairs mucus production/transport to the post-branchial digestive tract, and this makes the gut epithelium more vulnerable to microorganism colonisation. Regarding the effect of size and time of exposure to nanoplastics, we expect bigger particles to accumulate more easily in the gut after being trapped by mucus in the pharynx (8), thereby depleting the external nanoplastics concentration with time. In the memory experiments, the gut from primed animals reacts to an LPS challenge by increasing the expression of *VCBP* genes. This upregulation likely occurs in newly differentiating cells of the stomach. Since the stomach is the tract of the gut system with the fastest cell-renewing rate (about 14 days from stem cells to fully mature cells) (37), we can hypothesise that the stem cells primed by H/S and nanoplastics start expressing *VCBP* genes during the LPS-induced differentiation. Previous studies on *VCBP* expression in adult animals showed that *VCBP-C* is expressed in crypts, the stem cells reservoir, and in mucous cells, located at the junction between stomach and intestine, whereas *VCBP-B* is detected in folds, with a scattered pattern (38). Therefore, the potentiation of *VCBP-B* seems to represent a true memory-induced protective response, while the upregulation of *VCBP-C* may represent a sustained response, *i.e.*, a re-stimulation of a response already ongoing in developing stem cells.

The most striking findings in this study regard the regulation of the *VCBP* genes. Chitin-binding peptides/proteins and chitinase-like proteins are involved in constitutive and induced resistance to fungal colonisation, and are found in a number of organisms (39), including bacteria (40), plants (41), invertebrates (42, 43) and humans (44). The antifungal activity of these proteins is likely the result of their binding to nascent fungal cell wall chitin, through their chitin-binding lectin-like domain (CBD), resulting in disrupted fungal cell polarity with concomitant inhibition of growth (39). The variable region-containing chitin-binding domain proteins (VCBPs), thus far identified in two protochordates, the amphioxus *Branchiostoma floridae* (45) and the ascidian *Ciona robusta* (28), are secreted molecules expressed in stomach and gut lumen and in blood (28, 38). These proteins are thought to actively contribute to gut microbiota homeostasis *via* their IgV domains (46), while the Ig domains apparently have a structural role that allows for optimal binding to the cell walls, sporangia (spore-forming bodies) and spores of a diverse set of filamentous fungi isolated from the *Ciona* gut (47). The function of VCBPs secreted in the blood has been ascribed to their opsonising activity towards bacteria, mediated by the Ig domains with the contribution of CBD (27, 48). Since most studies focused on *VCBP* expression in the stomach and gut, there is no information about their expression and possible role in the pharynx, the filter feeding organ of the animal from which food particles entangled in mucus, secreted by the endostyle, are moved to the alimentary canal to be digested.

In our study, we have assessed the expression of *VCBP-B* and *VCBP-C* in the gut and in the pharynx of *C. robusta* in response to various stimuli, both as primary immune reaction and in immune memory responses. The *VCBP-B* gene product, expressed in the stomach of adults and in blood cells of *Ciona*, is a protein displaying one IgV domain, one Ig-like domain and one CBD. Conversely, the *VCBP-C* gene product, expressed in the stomach and gut of juvenile and adult animals and in blood cells, displays two Ig-like domains (no IgV domain) and a CBD. The differences in their expected functions are however not known (48). We observed that these genes are sensitive to every stimulus administered to animals, but in an organ-specific way. In response to systemic LPS, both genes were strongly up-regulated in the pharynx (over 4000-fold for *VCBP-B*), while in the gut the basal *VCBP-B* expression was decreased to zero in response to LPS and that of *VCBP-C* was not affected. Examining the gene expression in response to H/S stress, we observed a significant up-regulation of both genes (about 5-8-fold for *VCBP-B* and 200-fold for *VCBP-C*) upon both short and long stress. We could hypothesise that H/S stress decreases the mucus production and/or mucus transport (10) from the endostyle to the gut, and consequently the gut epithelium is less protected, resulting in an inflammatory condition similar to the experimental colitis in the mouse, which also displays a significant functional/physical impairment of the gut barrier (49). In the mouse colitis model, the disease is associated with an overgrowth of *Candida albicans* (50). Fungal overgrowth in the gut is in turn associated with dysbiotic changes in the gut microbiota and alterations of the digestive mucosa that promote *C. albicans* translocation across the digestive intestinal barrier and its haematogenous dissemination and invasive fungal infections (51, 52). Therefore, we can hypothesise that, following a stressful event compromising the mucus production/transport, the strong up-regulation of *VCBP* genes (mostly *VCBP-C* whose tissue expression pattern involves the majority of stomach outer folds and gut) could be due to the urgency of fighting epithelium colonisation by microorganisms. When animals are exposed to nanoplastics in the same H/S conditions, the up-regulation of *VCBP-C* was lost, similar to other genes, probably because of a higher toxicity of particles for the gut cells, poorly protected by mucus.

In the pharynx, LPS was extremely potent in up-regulating the expression of *VCBP-B*. So far, this gene was known to be expressed in the stomach of adult individuals and in granular amoebocytes (28, 38), but our qPCR data could also show an expression in the pharynx. However, the systemic LPS administration implies the stimulus reaching the blood, thus it is possible that granular amoebocytes contribute to the gene up-regulation observed in the pharynx. From our previous study (27), we know that the intratunical LPS administration in *Ciona* triggers an inflammatory response, which entails blood cell composition changes as well as a transcriptomic modulation of several genes. The strong induction of *VCBP-B* expression in the pharynx of LPS-treated animals is consistent with a close relationship between *VCBP*s and inflammatory environments and bacterial burden. Such correlation is supported by data in different systems. For instance, the human chitinase-3-like protein 1 (YKL-40) is highly expressed in serum from healthy volunteers inoculated with *Escherichia coli* endotoxin (53). In *S. pneumoniae*-infected mice, the Chi3l1 protein is up-regulated and plays central roles in promoting bacterial clearance and mediating host tolerance through inhibition of macrophage pyroptosis (54).

Although the exact functions of different VCBP proteins are still unknown (48), our data support the hypothesis that VCBPs exhibits a tissue-specific protective function, with VCBP-B mainly involved in patrolling the pharynx/blood district, whereas VCBP-C is mainly involved in the control of gut homeostasis.

In conclusion, our study demonstrates that *C. robusta* mounts a potent organ-specific adaptation response to environmental changes (the combination of hypoxia and starvation) and that such response depends on the duration of stress. The adaptation response is different in the pharynx and the gut or H/S-exposed animals, underlining the different protective role of the two organs. The presence of nanoplastics, in combination with H/S stress, changes gene expression as compared to H/S alone, resulting in an anomalous expression profile in the pharynx after 18 h of exposure and in a general inhibition of the H/S-induced changes in the gut. This may result in a hampered capacity of the animals to adapt to the environmental changes. When assessing the immune response to a bacterial challenge (LPS) in animals pre-exposed to H/S with or without nanoplastics, we could observe that the strong reaction to LPS in the pharynx was substantially decreased in animals that had previously experienced an H/S stress, whereas the low/down-regulated gene expression in the gut in response to LPS was generally abolished in previously stressed animals. Thus, animals experiencing environmental stress mount a robust adaptation response, based on changes in the expression of several immune/stress-related genes. Such adaptation (immune memory) strongly influences the immune reaction to a subsequent infectious challenge, which is less strong both in the gut and in the pharynx. We can hypothesise that the mitigation of the response to LPS (decrease in gene up-regulation in the pharynx and increase of gene down-regulation in the gut) may have a double effect, less protective capacity against infections but also less self-damage caused by an exceedingly strong reaction. It is notable that the presence of nanoplastics, which has effects on the primary adaptation response, does not have substantial effects on the H/S stress-induced memory reaction to ensuing challenges. This may be interpreted as an interference (likely due to particle accumulation in tissues) with the tissue homeostasis and functionality, *i.e.*, with adaptation to the environmental changes, which however does not substantially affect the defensive capacity (measured as memory response to an infectious challenge).

## Data availability statement

The original contributions presented in the study are included in the article/**Supplementary Material**. Further inquiries can be directed to the corresponding author.

## Ethics statement

Studies on *Ciona robusta* do not need ethical review and approval, in accordance with the national and international legislation regulating animal experimentation. All activities were performed according to the Italian DLgs 26/2014, the European Directive 2010/63/EU and the associated “A working document on Animal Welfare Bodies and National Committees to fulfil the requirements under the Directive”. Ethical clearance was confirmed by the Animal Welfare Committee of the Stazione Zoologica Anton Dohrn (Ethical Clearance Waive request 01/2023).

## Author contributions

RM and DM planned and performed the experiments. PI and DB contributed to the experimental design. All authors contributed to writing the manuscript. PI and DB critically revised the manuscript. All authors contributed to the article and approved the submitted version.
