# Supplementary material for: Environmental stress and nanoplastics’ effects on Ciona robusta: regulation of immune/stress-related genes and induction of innate memory in pharynx and gut
Source: Front Immunol. 2023 May 29;14:1176982. doi: 10.3389/fimmu.2023.1176982 (PMC10258323; doi:10.3389/fimmu.2023.1176982)
Supplement: Supplementary file 1 [file Table_1.docx]

**SUPPLEMENTARY TABLE S1.**

**Immune/stress-related gene expression in the pharynx and gut of *C. robusta* exposed to LPS or H/S stress**

| ***Gene*** | **Gene expression level ^a^** (mean ± SEM) | | | | | | | |
| --- | --- | --- | --- | --- | --- | --- | --- | --- |
|  | **Pharynx** | | | | **Gut** | | | |
|  | **Control** | **LPS** | **H/S 2 h** | **H/S 18 h** | **Control** | **LPS** | **H/S 2 h** | **H/S 18 h** |
| *C3-1* | 2.52 ± 1.49 | 4.54 ± 1.22 | 9.88 ± 1.7*^*^* | 15.5 ± 5.6*^*^* | 1.22 ± 0.50 | 0.10 ± 0.05 | 3.70 ± 0.90 | 69.8 ± 7.2*^*^*^#^ |
| *C3ar* | 2.29 ± 1.73 | 2.18 ± 0.59 | 7.35 ± 1.63 | 6.19 ± 2.57 | 1.04 ± 0.21 | 6.55 ± 1.08*^*^* | 55.4 ± 13.4*^**^* | 44.5 ± 6.9*^*^* |
| *Il17-1* | 1.16 ± 0.47 | 1.28 ± 0.38 | 0.17 ± 0.03 | 1.88 ± 0.03 | 1.08 ± 0.29 | 0.53 ± 0.21 | 3.44 ± 0.3 | 1.36 ± 0.21 |
| *Il17-2* | 1.76 ± 1.16 | 10.0 ± 3.7*^**^* | 3.81 ± 0.84 | 2.11 ± 0.11 | 1.49 ± 0.94 | 1.30 ± 0.19 | 5.89 ± 0.61*^*^* | 4.45 ± 1.68 |
| *Il17r* | 1.36 ± 0.61 | 1.37 ± 0.49 | 0.69 ± 0.06 | 15.2 ± 0.3*^*^*^#^ | 1.09 ± 0.33 | 0.03 ± 0.01*^*^* | 1.86 ± 0.26 | 34.9 ± 3.6*^*^*^#^ |
| *Tnf* | 1.19 ± 0.40 | 62.3 ± 5.7*^**^* | 6.90 ± 0.48*^*^* | 37.0 ± 3.8*^**^*^#^ | 1.18 ± 0.51 | 0.08 ± 0.02 | 2.32 ± 0.12 | 12.2 ± 1.7*^*^*^#^ |
| *Tgfb* | 1.06 ± 0.24 | 80.9 ± 9.5*^**^* | 9.09 ± 0.31*^*^* | 12.9 ± 1.3*^*^* | 2.12 ± 1.37 | 0.52 ± 0.24 | 16.9 ± 1.8*^*^* | 60.1 ± 1.0*^*^*^#^ |
| *Lbp* | 1.05 ± 0.23 | 1.90 ± 0.17 | 18.7 ± 2.9*^**^* | 16.3 ± 8.9*^*^* | 1.10 ± 0.35 | 0.31 ± 0.09 | 10.6 ± 0.7*^*^* | 3.45 ± 1.15 |
| *Tlr-2* | 1.31 ± 0.53 | 11.4 ± 1.4*^*^* | 1.84 ± 0.80 | 2.74 ± 0.19 | 1.08 ± 0.26 | 0.62 ± 0.01 | 8.11 ± 2.43*^*^* | 2.96 ± 0.61 |
| *Tlr13* | 1.04 ± 0.20 | 0.45 ± 0.14 | 0.58 ± 0.04 | 0.57 ± 0.25 | 1.35 ± 0.74 | 0.28 ± 0.04 | 2.33 ± 0.70 | 0.57 ± 0.25 |
| *Cd36* | 1.06 ± 0.24 | 0.59 ± 0.08 | 0.53 ± 0.12 | 0.35 ± 0.06 | 1.17 ± 0.49 | 0.15 ± 0.05 | 4.10 ± 2.17 | 10.5 ± 3.3*^*^* |
| *VCBP-B* | 1.11 ± 0.36 | 4129.5 ± 1042.5*^***^* | 6.23 ± 1.68*^*^* | 2.24 ± 0.31 | 1.25 ± 0.61 | 0.00 ± 0.00*^*^* | 9.76 ± 2.79*^*^* | 6.66 ± 0.80*^*^* |
| *VCBP-C* | 1.09 ± 0.27 | 30.2 ± 6.4*^**^* | 7.36 ± 1.01*^*^* | 1.20 ± 0.04^#^ | 1.13 ± 0.40 | 0.63 ± 0.09 | 187.8 ± 58.9*^**^* | 207.6 ± 42.6*^**^* |
| *SodA* | 1.04 ± 0.20 | 0.82 ± 0.59 | 2.40 ± 0.33 | 2.72 ± 0.42 | 1.43 ± 0.79 | 0.50 ± 0.15 | 9.79 ± 0.17*^*^* | 2.06 ± 0.32^#^ |
| *GST* | 1.06 ± 0.26 | 0.03 ± 0.00*^**^* | 0.16 ± 0.01 | 0.16 ± 0.05 | 2.43 ± 1.93 | 0.17 ± 0.02 | 2.15 ± 0.88 | 1.50 ± 0.10 |
| *GR* | 1.09 ± 0.33 | 3.05 ± 1.38 | 6.43 ± 2.34*^*^* | 4.10 ± 1.17 | 1.26 ± 0.51 | 0.06 ± 0.00*^*^* | 2.78 ± 1.97 | 4.00 ± 0.00 |

^a^ relative to *Gapdh*

*^*^*p<0.05, *^**^*p<0.01, *^***^*p<0.005 *vs*. control

^#^p<0.05, H/S 18 h *s*. 2 h

**SUPPLEMENTARY TABLE S2.**

**Immune/stress-related gene expression in the pharynx of *C. robusta* exposed to H/S stress**

| ***Gene*** | **Gene expression level ^a^** (mean ± SEM) | | | | | | |
| --- | --- | --- | --- | --- | --- | --- | --- |
|  | **Control** | **H/S 2 h** | **H/S 2 h**  **+ Npl 0.1 μm** | **H/S 2 h**  **+ Npl 0.35 μm** | **H/S 18 h** | **H/S 18 h**  **+ Npl 0.1 μm** | **H/S 18 h**  **+ Npl 0.35 μm** |
| *C3-1* | 2.52 ± 1.49 | 9.88 ± 1.7*^*^* | 3.22 ± 0.97^#^ | 2.15 ± 1.28^#^ | 15.5 ± 5.6*^*^* | 0.72 ± 0.09^#^ | 1.19 ± 0.46^#^ |
| *C3ar* | 2.29 ± 1.73 | 7.35 ± 1.63 | 4.76 ± 0.49 | 4.76 ± 0.52 | 6.19 ± 2.57 | 4.61 ± 1.74 | 25.5 ± 8.7*^*^*^#^ |
| *Il17-1* | 1.16 ± 0.47 | 0.17 ± 0.03 | 0.58 ± 0.05 | 0.57 ± 0.22 | 1.88 ± 0.03 | 0.48 ± 0.31 | 1.59 ± 0.55 |
| *Il17-2* | 1.76 ± 1.16 | 3.81 ± 0.84 | 4.13 ± 0.90 | 5.69 ± 1.56 | 2.11 ± 0.11 | 14.2 ± 5.8*^*^*^#^ | 18.6 ± 6.1*^*^*^#^ |
| *Il17r* | 1.36 ± 0.61 | 0.69 ± 0.06 | 0.38 ± 0.12 | 0.45 ± 0.15 | 15.2 ± 0.3 | 0.07 ± 0.03^#^ | 0.56 ± 0.07^#^ |
| *Tnf* | 1.19 ± 0.40 | 6.90 ± 0.48*^*^* | 13.1 ± 2.2 | 9.86 ± 2.15*^*^* | 37.0 ± 3.8*^**^* | 0.33 ± 0.22^##^ | 2.74 ± 0.92^##^ |
| *Tgfb* | 1.06 ± 0.24 | 9.09 ± 0.31*^*^* | 24.0 ± 3.3*^**^*^#^ | 22.5 ± 6.1*^**^*^#^ | 12.9 ± 1.3*^*^* | 7.89 ± 2.13*^*^* | 145.7 ± 46.4*^**^*^##^ |
| *Lbp* | 1.05 ± 0.23 | 18.7 ± 2.9*^**^* | 8.88 ± 1.12*^*^*^#^ | 12.8 ± 2.2*^*^* | 16.3 ± 8.9*^*^* | 8.27 ± 1.14*^*^* | 3.55 ± 1.18 |
| *Tlr-2* | 1.31 ± 0.53 | 1.84 ± 0.80 | 0.78 ± 0.04 | 3.96 ± 1.76 | 2.74 ± 0.19 | 2.26 ± 1.22 | 1.73 ± 0.6 |
| *Tlr13* | 1.04 ± 0.20 | 0.58 ± 0.04 | 0.75 ± 0.05 | 0.85 ± 0.36 | 0.57 ± 0.25 | 1.02 ± 0.66 | 4.32 ± 1.47*^*^*^#^ |
| *Cd36* | 1.06 ± 0.24 | 0.53 ± 0.12 | 1.40 ± 0.06 | 1.98 ± 0.97 | 0.35 ± 0.06 | 0.89 ± 0.35 | 6.89 ± 1.84*^*^*^#^ |
| *VCBP-B* | 1.11 ± 0.36 | 6.23 ± 1.68*^*^* | 6.80 ± 1.92*^*^* | 10.9 ± 3.7*^*^* | 2.24 ± 0.31 | 2.32 ± 0.20 | 36.2 ± 16.7*^**^*^#^ |
| *VCBP-C* | 1.09 ± 0.27 | 7.36 ± 1.01*^*^* | 7.56 ± 1.94*^*^* | 15.4 ± 3.72*^*^* | 1.20 ± 0.04 | 4.51 ± 3.31 | 9.51 ± 4.47*^*^*^#^ |
| *SodA* | 1.04 ± 0.20 | 2.40 ± 0.33 | 3.51 ± 0.58 | 1.30 ± 0.53 | 2.72 ± 0.42 | 0.71 ± 0.40 | 5.53 ± 1.53*^*^* |
| *GST* | 1.06 ± 0.26 | 0.16 ± 0.01 | 0.02 ± 0.00 | 0.05 ± 0.02 | 0.16 ± 0.05 | 0.02 ± 0.01 | 0.10 ± 0.04 |
| *GR* | 1.09 ± 0.33 | 6.43 ± 2.34*^*^* | 3.62 ± 0.43 | 2.01 ± 0.91 | 4.10 ± 1.17 | 1.27 ± 0.78 | 4.39 ± 1.68 |

^a^ relative to *Gapdh*

*^*^*p<0.05, *^**^*p<0.01, *vs*. control

^#^p<0.05, ^##^p<0.01, H/S + Nlp *vs*. H/S

**SUPPLEMENTARY TABLE S3.**

**Immune/stress-related gene expression in the gut of *C. robusta* exposed to H/S stress**

| ***Gene*** | **Gene expression level ^a^** (mean ± SEM) | | | | | | |
| --- | --- | --- | --- | --- | --- | --- | --- |
|  | **Control** | **H/S 2 h** | **H/S 2 h**  **+ Npl 0.1 μm** | **H/S 2 h**  **+ Npl 0.35 μm** | **H/S 18 h** | **H/S 18 h**  **+ Npl 0.1 μm** | **H/S 18 h**  **+ Npl 0.35 μm** |
| *C3-1* | 1.22 ± 0.50 | 3.70 ± 0.90 | 0.09 ± 0.02^#^ | 0.10 ± 0.03^#^ | 69.8 ± 7.2*^*^* | 0.13 ± 0.01^##^ | 0.08 ± 0.02^##^ |
| *C3ar* | 1.04 ± 0.21 | 55.4 ± 13.4*^**^* | 10.5 ± 3.0*^*^*^#^ | 4.76 ± 0.52*^*^*^#^ | 44.5 ± 6.9*^*^* | 8.12 ± 1.39*^*^*^#^ | 5.73 ± 1.24*^*^*^#^ |
| *Il17-1* | 1.08 ± 0.29 | 3.44 ± 0.30 | 3.26 ± 0.17 | 2.56 ± 0.19 | 1.36 ± 0.21 | 2.83 ± 1.46 | 1.94 ± 0.35 |
| *Il17-2* | 1.49 ± 0.94 | 5.89 ± 0.61*^*^* | 9.31 ± 1.85*^*^* | 5.04 ± 0.52*^*^* | 4.45 ± 1.68 | 4.52 ± 1.97 | 4.43 ± 1.12 |
| *Il17r* | 1.09 ± 0.33 | 1.86 ± 0.26 | 0.25 ± 0.02 | 0.42 ± 0.13 | 34.9 ± 3.6*^*^* | 0.35 ± 0.01^#^ | 0.61 ± 0.20^#^ |
| *Tnf* | 1.18 ± 0.51 | 2.32 ± 0.12 | 0.10 ± 0.06 | 0.16 ± 0.04 | 12.2 ± 1.7*^*^* | 1.19 ± 0.36^#^ | 0.16 ± 0.02^#^ |
| *Tgfb* | 2.12 ± 1.37 | 16.9 ± 1.8*^*^* | 10.3 ± 0.9*^*^*^#^ | 7.72 ± 2.25*^*^*^#^ | 60.1 ± 1.0*^*^* | 7.47 ± 1.41*^*^*^#^ | 5.22 ± 1.57^#^ |
| *Lbp* | 1.10 ± 0.35 | 10.6 ± 0.7*^*^* | 5.00 ± 0.87*^*^*^#^ | 0.53 ± 0.10^#^ | 3.45 ± 1.15 | 0.83 ± 0.40 | 0.48 ± 0.26 |
| *Tlr-2* | 1.08 ± 0.26 | 8.11 ± 2.43*^*^* | 1.71 ± 0.30^#^ | 1.26 ± 0.17^#^ | 2.96 ± 0.61 | 0.98 ± 0.17 | 0.58 ± 0.14 |
| *Tlr13* | 1.35 ± 0.74 | 2.33 ± 0.70 | 1.05 ± 0.13 | 0.43 ± 0.01 | 0.57 ± 0.25 | 0.56 ± 0.14 | 0.32 ± 0.08 |
| *Cd36* | 1.17 ± 0.49 | 4.10 ± 2.17 | 1.92 ± 0.23 | 2.40 ± 0.34 | 10.5 ± 3.3*^*^* | 0.96 ± 0.23^#^ | 1.35 ± 0.34^#^ |
| *VCBP-B* | 1.25 ± 0.61 | 9.76 ± 2.79*^*^* | 2.66 ± 1.08^#^ | 4.05 ± 0.59 | 6.66 ± 0.80*^*^* | 3.48 ± 0.12 | 1.89 ± 0.62^#^ |
| *VCBP-C* | 1.13 ± 0.40 | 187.8 ± 58.9*^**^* | 11.0 ± 2.9*^*^*^##^ | 8.78 ± 2.84*^*^*^##^ | 207.6 ± 42.6*^**^* | 9.46 ± 1.46*^*^*^##^ | 3.02 ± 0.92^##^ |
| *SodA* | 1.43 ± 0.79 | 9.79 ± 0.17*^*^* | 5.33 ± 1.06*^*^* | 6.56 ± 1.58*^*^* | 2.06 ± 0.32 | 3.92 ± 0.27 | 3.38 ± 0.53 |
| *GST* | 2.43 ± 1.93 | 2.15 ± 0.88 | 0.48 ± 0.11 | 1.09 ± 0.03 | 1.50 ± 0.10 | 0.47 ± 0.14 | 0.89 ± 0.15 |
| *GR* | 1.26 ± 0.51 | 2.78 ± 1.97 | 2.31 ± 0.52 | 2.95 ± 0.74 | 4.00 ± 0.00 | 8.49 ± 1.02*^*^* | 3.01 ± 0.83 |

^a^ relative to *Gapdh*

*^*^*p<0.05, *^**^*p<0.01, *^***^*p<0.005, *vs*. control

^#^p<0.05, ^##^p<0.01, H/S + Nlp *vs*. H/S

**SUPPLEMENTARY TABLE S4.**

**Nanoplastics-dependent modulation of H/S-induced immune memory response to LPS in the pharynx of *C. robusta***

| ***Gene*** | **Gene expression level ^a^** (mean ± SEM) | | | | | | | |
| --- | --- | --- | --- | --- | --- | --- | --- | --- |
|  | **no pre-exposure** | | **pre-exposed 2 h to H/S +** | | | **pre-exposed 18 h to H/S +** | | |
|  |  |  | **no Npl** | **Npl 0.1 μm** | **Npl 0.35 μm** | **no Npl** | **Npl 0.1 μm** | **Npl 0.35 μm** |
|  | **No challenge** | **Challenged with LPS** | | | | | | |
| *C3-1* | 2.52 ± 1.49 | 4.54 ± 1.22 | 1.82 ± 1.14 | 0.58 ± 0.23 | 5.96 ± 0.49 | 2.32 ± 0.60 | 2.03 ± 0.13 | 2.75 ± 0.67 |
| *C3ar* | 2.29 ± 1.73 | 2.18 ± 0.59 | 2.13 ± 1.30 | 13.5 ± 6.0^*#^ | 6.77 ± 3.58 | 3.57 ± 1.08 | 3.89 ± 1.16 | 13.1 ± 4.2^*#^ |
| *Il17-1* | 1.16 ± 0.47 | 1.28 ± 0.38 | 0.16 ± 0.07 | 0.15 ± 0.01 | 0.26 ± 0.10 | 0.09 ± 0.02 | 0.31 ± 0.09 | 0.27 ± 0.08 |
| *Il17-2* | 1.76 ± 1.16 | 10.0 ± 3.7^**^ | 1.15 ± 0.23 | 2.63 ± 0.39 | 4.09 ± 0.60 | 2.63 ± 0.21 | 3.74 ± 0.11 | 5.53 ± 1.88 |
| *Il17r* | 1.36 ± 0.61 | 1.37 ± 0.49 | 0.29 ± 0.11 | 1.13 ± 0.16 | 1.00 ± 0.12 | 1.07 ± 0.24 | 1.77 ± 0.51 | 1.11 ± 0.27 |
| *Tnf* | 1.19 ± 0.40 | 62.3 ± 5.7^**^ | 5.22 ± 2.31^*^^ | 23.4 ± 3.3^*#^ | 30.6 ± 1.7^*#^ | 6.20 ± 1.34^*^^ | 18.3 ± 5.2^*^#^ | 38.3 ± 4.1^*#^ |
| *Tgfb* | 1.06 ± 0.24 | 80.9 ± 9.5^**^ | 18.4 ± 6.8^*^^ | 103.6 ± 28.2^**#^ | 80.7 ± 14.0^**#^ | 15.2 ± 4.4^*^^ | 10.9 ± 3.8^*^^ | 46.1 ± 12.4^**#^ |
| *Lbp* | 1.05 ± 0.23 | 1.90 ± 0.17 | 1.74 ± 0.92 | 11.9 ± 4.1^*^#^ | 30.7 ± 6.3^*^#^ | 3.53 ± 1.34 | 14.8 ± 3.0^*^#^ | 9.10 ± 2.34^*^^ |
| *Tlr-2* | 1.31 ± 0.53 | 11.4 ± 1.4^*^ | 0.70 ± 0.29^^^ | 0.65 ± 0.15^^^ | 1.07 ± 0.42^^^ | 0.88 ± 0.11^^^ | 0.95 ± 0.20^^^ | 1.16 ± 0.32^^^ |
| *Tlr13* | 1.04 ± 0.20 | 0.45 ± 0.14 | 1.36 ± 0.50 | 0.96 ± 0.03 | 1.23 ± 0.34 | 1.30 ± 0.14 | 0.83 ± 0.29 | 1.50 ± 0.33 |
| *Cd36* | 1.06 ± 0.24 | 0.59 ± 0.08 | 1.61 ± 0.76 | 1.59 ± 0.17 | 2.01 ± 0.40 | 3.05 ± 0.65 | 2.22 ± 0.83 | 4.66 ± 2.16 |
| *VCBP-B* | 1.11 ± 0.36 | 4129.5 ± 1042.5^**^ | 2.36 ± 1.46^^^^ | 20.8 ± 3.9^*^^#^ | 58.4 ± 12.4^*^^#^ | 9.29 ± 3.06^*^^^ | 47.6 ± 3.0^*^^#^ | 28.7 ± 4.0^*^^#^ |
| *VCBP-C* | 1.09 ± 0.27 | 30.2 ± 6.4^*^ | 2.10 ± 0.67^^^ | 4.80 ± 0.93^*^^ | 1.39 ± 0.36^^^ | 2.92 ± 1.61^^^ | 0.92 ± 0.20^^^ | 3.09 ± 1.34^^^ |
| *SodA* | 1.04 ± 0.20 | 0.82 ± 0.59 | 1.39 ± 0.64 | 3.23 ± 0.70 | 7.65 ± 3.44^*^#^ | 1.39 ± 0.31^#^ | 1.29 ± 0.03 | 3.17 ± 1.67 |
| *GST* | 1.06 ± 0.26 | 0.03 ± 0.00^**^ | 0.89 ± 0.35 | 2.07 ± 0.49 | 6.19 ± 1.57^*^#^ | 0.51 ± 0.08 | 0.85 ± 0.41 | 4.19 ± 0.81^^^ |
| *GR* | 1.09 ± 0.33 | 3.05 ± 1.38 | 1.64 ± 0.87 | 4.29 ± 0.85 | 4.76 ± 0.83 | 3.50 ± 0.26 | 4.88 ± 1.55 | 8.37 ± 1.91^*^ |

^a^ relative to *Gapdh*

*^*^*p<0.05, *^**^*p<0.01, *^***^*p<0.005, challenged with LPS *vs*. no challenge

^^^p<0.05, ^^^^p<0.01, pre-exposed to H/S *vs*. no pre-exposure (all challenged with LPS)

^#^p<0.05, pre-exposed to H/S + Nlp *vs*. H/S (all challenged with LPS)

**SUPPLEMENTARY TABLE S5.**

**Nanoplastics-dependent modulation of H/S-induced immune memory response to LPS in the gut of *C. robusta***

| ***Gene*** | **Gene expression level ^a^** (mean ± SEM) | | | | | | | |
| --- | --- | --- | --- | --- | --- | --- | --- | --- |
|  | **no pre-exposure** | | **pre-exposed 2 h to H/S +** | | | **pre-exposed 18 h to H/S +** | | |
|  |  |  | **no Npl** | **Npl 0.1 μm** | **Npl 0.35 μm** | **no Npl** | **Npl 0.1 μm** | **Npl 0.35 μm** |
|  | **No challenge** | **Challenged with LPS** | | | | | | |
| *C3-1* | 1.22 ± 0.50 | 0.10 ± 0.05 | 0.66 ± 0.33 | 0.05 ± 0.04 | 0.15 ± 0.06 | 0.18 ± 0.07 | 0.05 ± 0.01 | 0.74 ± 0.05 |
| *C3ar* | 1.04 ± 0.21 | 6.55 ± 1.08*^*^* | 3.01 ± 1.08 | 7.23 ± 0.88*^*^* | 4.03 ± 0.76 | 4.94 ± 1.57 | 7.92 ± 1.54*^*^* | 13.5 ± 1.5*^*^*^#^ |
| *Il17-1* | 1.08 ± 0.29 | 0.53 ± 0.21 | 0.49 ± 0.13 | 0.30 ± 0.18 | 0.61 ± 0.53 | 0.31 ± 0.03 | 0.68 ± 0.16 | 3.41 ± 1.03 |
| *Il17-2* | 1.49 ± 0.94 | 1.30 ± 0.19 | 2.18 ± 0.17 | 0.97 ± 0.25 | 0.86 ± 0.25 | 1.05 ± 0.47 | 1.38 ± 0.73 | 2.45 ± 1.42 |
| *Il17r* | 1.09 ± 0.33 | 0.03 ± 0.01*^*^* | 1.28 ± 0.26^^^ | 0.43 ± 0.21 | 0.84 ± 0.11^^^ | 0.71 ± 0.09^^^ | 0.96 ± 0.31^^^ | 1.12 ± 0.37^^^ |
| *Tnf* | 1.18 ± 0.51 | 0.08 ± 0.02 | 1.61 ± 0.47 | 0.50 ± 0.27 | 0.58 ± 0.43 | 0.34 ± 0.17 | 0.58 ± 0.17 | 0.74 ± 0.26 |
| *Tgfb* | 2.12 ± 1.37 | 0.52 ± 0.24 | 4.18 ± 1.83 | 12.9 ± 3.1*^*^*^^#^ | 2.94 ± 0.36 | 1.57 ± 0.86 | 6.66 ± 0.20^^#^ | 22.6 ± 2.2*^*^*^^#^ |
| *Lbp* | 1.10 ± 0.35 | 0.31 ± 0.09 | 0.90 ± 0.13 | 0.76 ± 0.46 | 0.38 ± 0.18 | 0.36 ± 0.04 | 0.37 ± 0.02 | 8.95 ± 0.84*^*^*^^#^ |
| *Tlr-2* | 1.08 ± 0.26 | 0.62 ± 0.01 | 1.52 ± 0.25 | 0.39 ± 0.11 | 0.59 ± 0.20 | 0.52 ± 0.22 | 0.59 ± 0.21 | 1.95 ± 0.56 |
| *Tlr13* | 1.35 ± 0.74 | 0.28 ± 0.04 | 1.35 ± 0.26 | 0.51 ± 0.27 | 0.43 ± 0.18 | 0.36 ± 0.14 | 0.80 ± 0.33 | 0.72 ± 0.20 |
| *Cd36* | 1.17 ± 0.49 | 0.15 ± 0.05 | 2.49 ± 0.25 | 1.26 ± 0.74 | 0.43 ± 0.24 | 0.41 ± 0.03 | 0.85 ± 0.23 | 0.95 ± 0.19 |
| *VCBP-B* | 1.25 ± 0.61 | 0.00 ± 0.00*^*^* | 3.01 ± 0.21^^^^ | 1.73 ± 0.56^^^ | 4.53 ± 1.01^^^^ | 1.96 ± 0.59^^^ | 8.03 ± 2.28*^*^*^^^#^ | 1.68 ± 0.79^^^ |
| *VCBP-C* | 1.13 ± 0.40 | 0.63 ± 0.09 | 6.80 ± 1.05*^*^*^^^ | 3.75 ± 0.51 | 5.51 ± 2.72*^*^*^^^ | 1.51 ± 0.79 | 13.0 ± 3.9*^*^*^^#^ | 2.14 ± 0.57 |
| *SodA* | 1.43 ± 0.79 | 0.50 ± 0.15 | 0.95 ± 0.41 | 0.79 ± 0.69 | 0.32 ± 0.15 | 0.11 ± 0.05 | 1.60 ± 1.23 | 1.89 ± 0.08 |
| *GST* | 2.43 ± 1.93 | 0.17 ± 0.02 | 1.25 ± 0.50 | 0.34 ± 0.12 | 1.02 ± 0.37 | 1.28 ± 0.44 | 0.36 ± 0.09 | 1.87 ± 0.94 |
| *GR* | 1.26 ± 0.51 | 0.06 ± 0.00*^*^* | 0.59 ± 0.27 | 1.46 ± 0.39^^^ | 1.44 ± 0.30^^^ | 0.22 ± 0.11 | 1.91 ± 1.05^^^ | 3.20 ± 0.89^^#^ |

^a^ relative to *Gapdh*

*^*^*p<0.05, no challenge *vs*. challenged with LPS

^^^p<0.05, ^^^^p<0.01, pre-exposed to H/S *vs*. no pre-exposure (all challenged with LPS)

^#^p<0.05, pre-exposed to H/S + Nlp *vs*. H/S (all challenged with LPS)
